# A new *Zymomonas mobilis* platform strain for the efficient production of chemicals

**DOI:** 10.1186/s12934-024-02419-9

**Published:** 2024-05-22

**Authors:** Jonas Frohwitter, Gerrich Behrendt, Steffen Klamt, Katja Bettenbrock

**Affiliations:** Analysis and Redesign of Biological Networks, Max Planck Institute for Dynamics of Complex Technical Systems, Sandtorstr. 1, 39106 Magdeburg, Germany

**Keywords:** *Zymomonas mobilis*, Platform strain, Metabolic engineering, Lactate, Alanine, Pyruvate decarboxylase

## Abstract

**Background:**

*Zymomonas mobilis* is well known for its outstanding ability to produce ethanol with both high specific productivity and with high yield close to the theoretical maximum. The key enzyme in the ethanol production pathway is the pyruvate decarboxylase (PDC) which is converting pyruvate to acetaldehyde. Since it is widely considered that its gene *pdc* is essential, metabolic engineering strategies aiming to produce other compounds derived from pyruvate need to find ways to reduce PDC activity.

**Results:**

Here, we present a new platform strain (sGB027) of *Z. mobilis* in which the native promoter of *pdc* was replaced with the IPTG-inducible P_T7A1,_ allowing for a controllable expression of *pdc*. Expression of lactate dehydrogenase from *E. coli* in sGB027 allowed the production of D-lactate with, to the best of our knowledge, the highest reported specific productivity of any microbial lactate producer as well as with the highest reported lactate yield for *Z. mobilis* so far. Additionally, by expressing the L-alanine dehydrogenase of *Geobacillus stearothermophilus* in sGB027 we produced L-alanine, further demonstrating the potential of sGB027 as a base for the production of compounds other than ethanol.

**Conclusion:**

We demonstrated that our new platform strain can be an excellent starting point for the efficient production of various compounds derived from pyruvate with *Z. mobilis* and can thus enhance the establishment of this organism as a workhorse for biotechnological production processes.

**Supplementary Information:**

The online version contains supplementary material available at 10.1186/s12934-024-02419-9.

## Background

*Zymomonas mobilis* is a facultative anaerobic Gram-negative bacterium and one of the most efficient microbial ethanol producers known [1]. While the wild type only has a narrow substrate spectrum, being able to metabolize glucose, fructose and sucrose, it has many desirable characteristics for use as an industrial biocatalyst [2]. It exhibits a broad pH spectrum (pH 3.5–7.5), tolerance to high sugar and ethanol concentrations, a very high ethanol yield (up to 98% of the maximum yield on glucose) and is generally regarded as safe (GRAS) [3, 4]. Moreover, as a facultative anaerobe, it is well suited for large-scale fermentation making precise aeration control obsolete. Unlike yeast or *Escherichia coli*, which use the Embden–Meyerhof–Parnas pathway for utilization of sugars, *Z. mobilis* uses the Entner − Doudoroff pathway which allows for a higher flux and thus a glucose uptake rate that is approximately 3–4 times higher than in the organisms mentioned above [4–7]. The high glycolytic flux is strongly linked to ethanol production, which is beneficial for high ethanol yield and productivity but hampers the production of other chemicals of interest. There is an increasing spectrum of products synthesized by genetically modified *Z. mobilis* strains, including malate, lactate, alanine, acetoin or the heterologous products 2,3-butanediol and isobutanol, but ethanol remains an essential by-product of all those strains, thereby reducing the yields for the respective product to varying degrees [8–15].

To reduce the amount of ethanol as a by-product, the activity of the enzyme pyruvate decarboxylase (PDC; GenBank: AAV89984.1), which converts pyruvate to acetaldehyde, has to be eliminated or strongly reduced. However, a complete knock-out of *pdc* (ZMO1360) in the wild type (WT) background proved impossible leading to different strategies to reduce its activity [8, 14–17]. Most studies were only able to generate heterogeneous knockout strains, which retained significant PDC activity. As a cornerstone, Liu et al. [15] were able to construct a knockout of the genomic copy of *pdc* in the presence of a plasmid-encoded copy expressed from an IPTG-inducible promoter, allowing for a variable *pdc* expression depending on the IPTG levels. Without induction, PDC activity was reduced 15-fold compared to the wild type. To account for the redox imbalance caused by the lack of ethanol production, other pathways converting pyruvate towards alternative products (2,3-butanediol, isobutanol, lactate) were introduced by overexpressing the corresponding enzymes. For example, expression of the lactate dehydrogenase from *E. coli*, resulted in lactate production of about 70% of the theoretical maximum [15] which is significantly higher than lactate production achieved in the wild type background with lactate dehydrogenase expressed either from a plasmid or integrated into the chromosome (20% and 15% of the theoretical maximum, respectively) [9, 14]. A complete removal of the *pdc* gene was described in a patent US 20190153483A1 [18]. However, a clean deletion could only be obtained after introduction of genes allowing for the production of 2,3-butanediol. As a result, this strain is optimized for 2,3-butanediol synthesis but cannot be used for the production of alternative products.

While the work of Liu et al. [15] was a key milestone on the way to turn *Z. mobilis* into a highly productive platform organism for synthesis of various products, further progress and improvements are needed. Moreover, the tunable *pdc* expression of the strains constructed in [15] is dependent on plasmids which could cause problems with plasmid stability or interfere with the expression of further heterologous genes. Therefore, in this work, we present a new chassis strain in which we replaced the native promoter of the chromosomal *pdc* gene with the IPTG-inducible promoter P_T7A1_. Heterologous expression of lactate dehydrogenase from *E. coli* or of the alanine dehydrogenase of *Geobacillus stearothermophilus* in this strain led to production of lactate and alanine, respectively, with high product yields and unprecedented specific productivities even in chemically defined media. These results demonstrate the enormous potential of our chassis strain as a new *Z. mobilis* platform strain for production of various chemicals.

## Materials and methods

### Strains and media

*Escherichia coli* strain NEB5α (New England Biolabs Inc, USA) was used for plasmid construction and maintenance and was cultivated in LB media (10 g/L tryptone, 5 g/L yeast extract, 5 g/L NaCl) at 37 °C. *Z. mobilis* wild type strain ZM4 (ATCC 31821) was used for the construction of sGB027. *Z. mobilis* pre-cultures as well as cultures and plates for strain construction were cultivated in Zymomonas complex medium (ZCM; bacto peptone 10 g/L, yeast extract 10 g/L, glucose 20 g/L; DSMZ GmbH) at 30 °C in closed flasks. Seed and main cultures were cultivated in Zymomonas minimal medium (ZMM) containing 1 g/L K_2_HPO_4_, 1 g/L KH_2_PO_4_, 0.5 g/L NaCl, 1 g/L NH_4_SO_4_, 0.2 g/L MgSO_4_·7H_2_O, 25 mg/L Na_2_MoO_4_·2H_2_O, 2.5 mg/L FeSO_4_·7H_2_O, 20 mg/L CaCl_2_·2H_2_O, 2 g/L Ca(HCO_3_)_2_, 1 mg/L calcium pantothenate, 20 g/L glucose (modified from [19]). If necessary, 100 µg/L kanamycin or 200 µg/L spectinomycin were added.

### Construction of plasmids

Plasmids were constructed using GoldenGate Cloning [20] based on our Zymo-Parts Toolbox [9]. DNA amplification by PCR was carried out according to the manufacturer’s recommendations using Q5 DNA Polymerase (New England Biolabs Inc., USA). All primers and assembly procedures are listed in Additional File 1. Native BsaI and BbsI recognition sites were removed to generate fragments compatible with the Zymo-Parts toolbox [9], retaining the original amino acid sequences of the proteins. For the shuttle vectors expressing either the alanine dehydrogenase from *G. stearothermophilus* (GenBank: EF154460.1) or the lactate dehydrogenase from *E. coli* (*ldhA*, GenBank: MCV5771625.1) a replication system based on pZMOB06 (Genbank: NC_017185) was used and P_strong100k*_, rbs_10k_ and T_soxR_ were used as regulatory elements. The gene sequence of the alanine dehydrogenase was codon optimized to fit the codon usage of *Z. mobilis*, by replacing the 22 codons that would have less than 15% usage with more frequently used codons based on the Kazusa codon usage database [21] entry for *Z. mobilis* subsp. *mobilis* ZM4.

All related DNA sequences can be accessed as annotated gene bank files in an online repository (10.17617/3.UM0Q7A).

### Construction of strains

Replacement of the native *pdc* promoter by the IPTG-inducible promoter P_T7A1_ [22] in the *Z. mobilis* chromosome was achieved by homologous recombination using the suicide vector pZP950. Homology arms adjacent to the P_*pdc*_ on the chromosome were chosen to be 800 bp long and were flanking a spectinomycin resistance cassette as well as an expression cassette for *lacI* and the LacI-regulated promoter P_T7A1_ [22]. *Z. mobilis* ZM4 was transformed with pZP950 via electroporation as previously described [9] and selection was carried out using 200 µg/ml spectinomycin on ZCM plates in the presence of 1 mM IPTG. Resulting colonies were tested on ZCM plates with and without 1 mM IPTG and colonies that only grew in the presence of IPTG were tested by colony PCR (see Additional File 1 for primer sequences) using OneTaq Quick-Load 2X Master Mix (New England Biolabs Inc., USA). The resulting homogenous strain with P_*pdc*_ replaced by P_T7A1_ was named sGB027 (ZM4 ΔP_*pd*_ :: *spR*-*lacI*-P_T7A1_).

Plasmids for either lactate dehydrogenase or alanine dehydrogenase expression were introduced into sGB027, resulting in strains sGB029 and sGB038, respectively.

### Lactate production

For lactate production, sGB029 pre-cultures and seed cultures were grown in minimal medium (ZMM) with IPTG and 100 µg/mL kanamycin according to [9]. Anaerobic main cultures were conducted in either standing cultures in 50 ml tubes with 50 ml minimal medium (ZMM) with 0–100 µM IPTG without pH control or in a batch fermentation with ZMM or complex medium (ZCM) with 100 µg/mL kanamycin in Infors HT Multifors 1.4 L fermenters (Infors AG, Switzerland) with the following setup: 400 mL working volume, 30 °C, stirrer speed 550 rpm, no aeration, pH 6.5 (kept by automated addition of 5 M NaOH). Fermentation parameters were monitored and controlled using the latest version of the eve software (Infors AG, Switzerland).

### Alanine production

Alanine production with strain sGB038 was conducted as described for lactate production. Since alanine production requires larger amounts of ammonium, 12.5 mM ammonium chloride was spiked to the fermentation medium every three hours by adding 2.5 mL of a 2 M ammonium chloride solution to the 400 mL working volume. For a typical experiment with about 24 h runtime this meant seven spikes and a total of 87.5 mM ammonium chloride.

### Analytics

Extracellular glucose and ethanol from experiments conducted in defined minimal medium (ZMM) were quantified via HPLC using an Agilent 1100 series system (Agilent Technologies, Germany) equipped with a Rezex-ROA column (Phenomenex, USA). 2 µl of the samples were injected onto the column by an autosampler and analyzed using isocratic elution with 4 mM H_2_SO_4_ at a flow rate of 0.5 mL/min at 60 °C and was detected with an RID detector. Extracellular lactate and alanine were quantified with an Inertsil ODS-3 column (GL Sciences, Germany). 10 µl of the samples were injected onto the column by an autosampler and analyzed using isocratic elution with 0.1 M ammonium phosphate monobasic (pH 2.6) at a flow rate of 0.1 mL/min at 40 °C and was detected with a DAD detector. Concentrations were calculated based on the peak area using standard solutions. For experiments conducted in undefined complex medium (ZCM), extracellular glucose, ethanol and lactate were quantified using the respective enzymatic assay kits from Megazyme (Ireland).

### Calculation of growth rate and specific uptake and production rates

Measured concentrations of biomass and metabolites (glucose, ethanol, lactate or alanine, respectively) in the medium were used to calculate the corresponding growth rate $\mu$ and specific excretion and uptake rates ${r}_{M}$ according to [23]. All rates and yields were first determined for each replicate and the means and standard deviations were calculated afterwards.

For exponentially growing cells, $\mu$ was determined by plotting the natural logarithm of the biomass concentrations of each sampled time point (within the exponential growth phase) against the cultivation time. The slope of the linear regression equals $\mu$ in ${\text{h}}^{\text{-1}}$. Excretion and uptake rates during exponential growth were calculated with the formula

$${r_M} = \mu \cdot \frac{{{c_{M,e}} - {c_{M,s}}}}{{{X_e} - {X_s}}}\left[ {{\text{mmol}} \cdot {\text{gCD}}{{\text{W}}^{{\text{ - 1}}}} \cdot {{\text{h}}^{{\text{ - 1}}}}} \right]$$

where $\mu$ is the growth rate, ${c}_{M,e}$ and ${c}_{M,s}$ are the end and start concentration of the respective metabolite M (in mM), and ${X}_{e}$ and ${X}_{s}$ represent the end and start concentration of biomass (in gCDW L^− 1^).

In one scenario we observed linear growth and calculated the linear growth rate ${\mu }_{lin}$(in gCDW/h) as ${\mu }_{lin}=\frac{{X}_{e}-{X}_{s}}{\varDelta t}$ where $\varDelta t={t}_{e}-{t}_{s}$ is the length of the time period (in h). For linear growth and for non-growing cells, excretion and uptake rates were calculated with the formula

$${r_M} = \frac{{{c_{M,e}} - {c_{M,s}}}}{{{X_{Av}} \cdot {\text{ }}\Delta t}}\left[ {{\text{mmol }} \cdot {\text{gCD}}{{\text{W}}^{{\text{ - 1}}}} \cdot {{\text{h}}^{{\text{ - 1}}}}} \right]$$

where ${X}_{Av}$ is the average biomass concentration (in gCDW L^− 1^) within the respective time period.

Product yields *Y*_P/S_ were determined by dividing *C*_*product, e*_ (in mmol) by *C*_*glucose, s*_ (in mmol).

Dry cell weight was deduced from OD600 measurements using a factor of 0.253 (OD600 of 1 equals 0.253 g CDW/L). This factor was determined experimentally in our laboratory by harvesting and weighting cells.

### Determination of PDC activity and expression

PDC activity was determined essentially as described by [15]. ZM4 and sGB027 were grown in ZMM with 2% glucose. 50 µM IPTG was added to sGB027 precultures. Precultures were washed in ZMM and inoculated to an OD600 of about 0.15–0.2. After 6–7 h when growth rate was constant, aliquots of cells corresponding to 1 OD of cells was harvested and the pellet was immediately frozen at -20 °C. The pellet was lysed by incubating it in 150 µl BugBuster Protein Extraction Reagent (EMD Millipore, USA) for 20 to 30 min at room temperature. Afterwards cell debris was removed by centrifugation. The protein concentration was determined using the Quibit protein BR reagent and the Qubit 4 fluorometer (Thermo Fisher Scientific, USA). The supernatants were diluted 1:10 or 1:25 in PBS and 5 µl were mixed with 195 µl of assay mixture containing 180 mM Na-citrate, pH 6.0, 25 mM pyruvate, 250 µM NADH and 10 U/ml alcohol dehydrogenase from yeast. Changes in the absorption at 340 nm were monitored in a PowerWave XS reader (BioTek Instruments, USA). PDC activity was determined as the rate of NADH oxidation per µg of protein.

For quantification of *pdc* transcript levels based on qRT-PCR [24, 25] about 1.5 × 10^9^ cells of sGB027 and ZM4 grown as described above were quenched in twice the volume of RNAprotect Bacterial Reagent (Qiagen, Germany), vortexed for 5 s and incubated at room temperature for 5 min. Cells were pelleted by centrifugation, the supernatant was discarded and the pellet was stored at − 80 °C. RNA preparation and qPCR were performed essentially as described previously [24]. RNA was prepared using the Master Pure RNA Purification Kit (Epicenter, USA). RNA concentration was determined using the NanoDrop spectrophotometer (Thermo Fisher Scientific, USA). mRNA was transcribed into cDNA by using the RevertAid H Minus First Strand cDNA synthesis Kit (Thermo Fisher Scientific, USA). Quantitative PCR of cDNA samples from two independent experiments was performed using the Takyon reagent (Eurogenetec, Belgium) with SYBR Green as detection agent and the Rotor-Gene 6000 (Qiagen, Germany). Amplification conditions were: 95 °C for 10 min, 40 cycles at 95 °C for 15 s and 60 °C for 1 min. A negative control without template was conducted for each primer pair and a control for DNA contamination was performed for each RNA sample used. Quantification was performed by relative quantification to the housekeeping genes (*gap* and *zwf*) applying the ΔΔCt method [26, 27] using the R package ddCt [28]. Besides the transcript levels of *pdc* we also determined the levels of *glk*, another housekeeping gene. Primer sequences are indicated in Supplemental File 1.

## Results

### Construction and characterization of the chassis strain sGB027

**Fig. 1:**
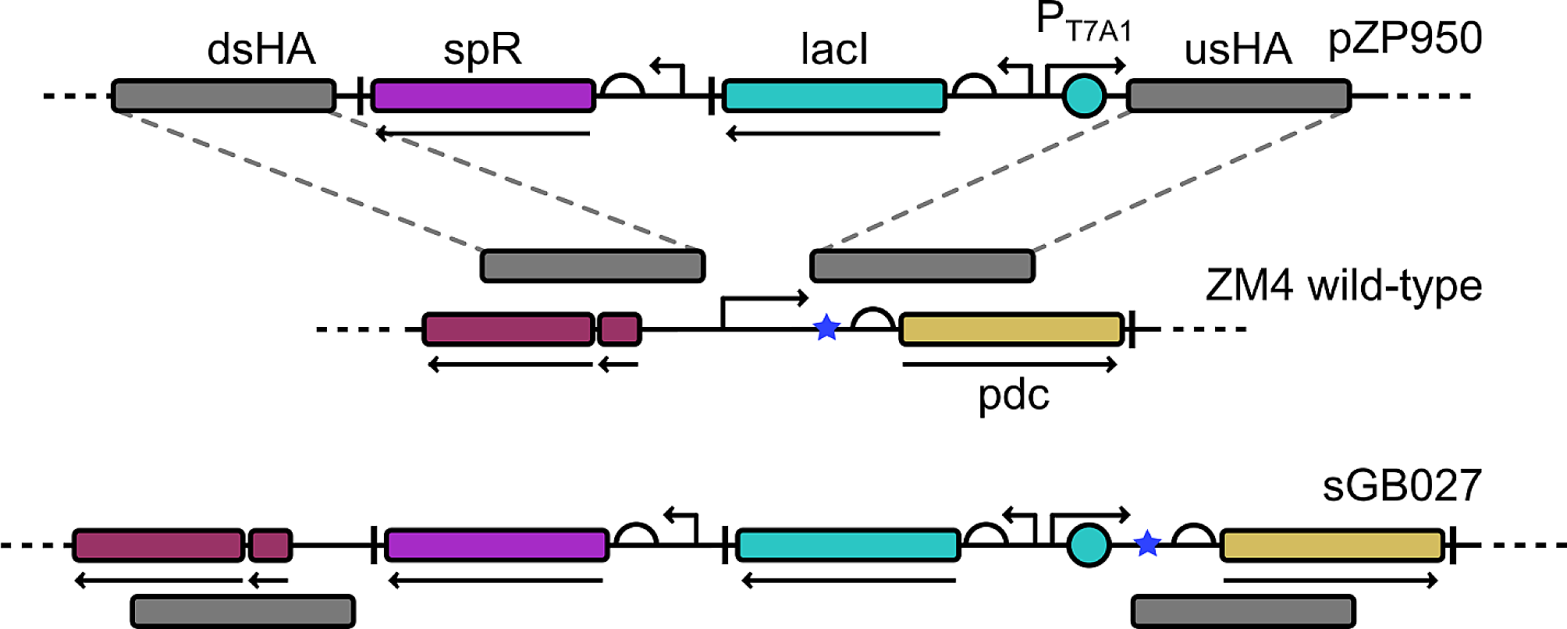
Graphical overview of the construction of strain sGB027. Top showing parts of pZP950, middle showing section of wildtype chromosome and bottom showing the same section in the edited strain sGB027. Promoters are indicated as perpendicular angled arrows, rbs as half circles and lac operator as azure circle. TSSs are indicated as blue stars, terminators as perpendicular lines, and ORFs as arrows under the elements. lacI is shown in azure, spectinomycin resistance in purple, pdc in yellow, homology arms in grey and other chromosomal genes in red

As explained in the Introduction section, using *Z. mobilis* for the efficient production of chemicals derived from pyruvate other than ethanol necessitates the reduction of PDC activity. We therefore exchanged the strong natural promoter P_pdc_ with the IPTG-inducible promoter P_T7A1_ to allow for a controlled expression of *pdc*. The homology arms for homologous recombination were selected based on the position of the five potential transcription start sites (TSS_3112 to TSS_3116) of the *pdc* gene [29] in order to replace the *pdc* promoter but to keep the native translation signals. Using homologous recombination with suicide vector pZP950, a fragment encoding a spectinomycin resistance cassette, a *lacI* expression cassette and the promoter P_T7A1_ [22] was inserted upstream of the *pdc* transcription start sites thereby replacing the native promoter to create strain sGB027 (see Fig. 1).

**Fig. 2:**
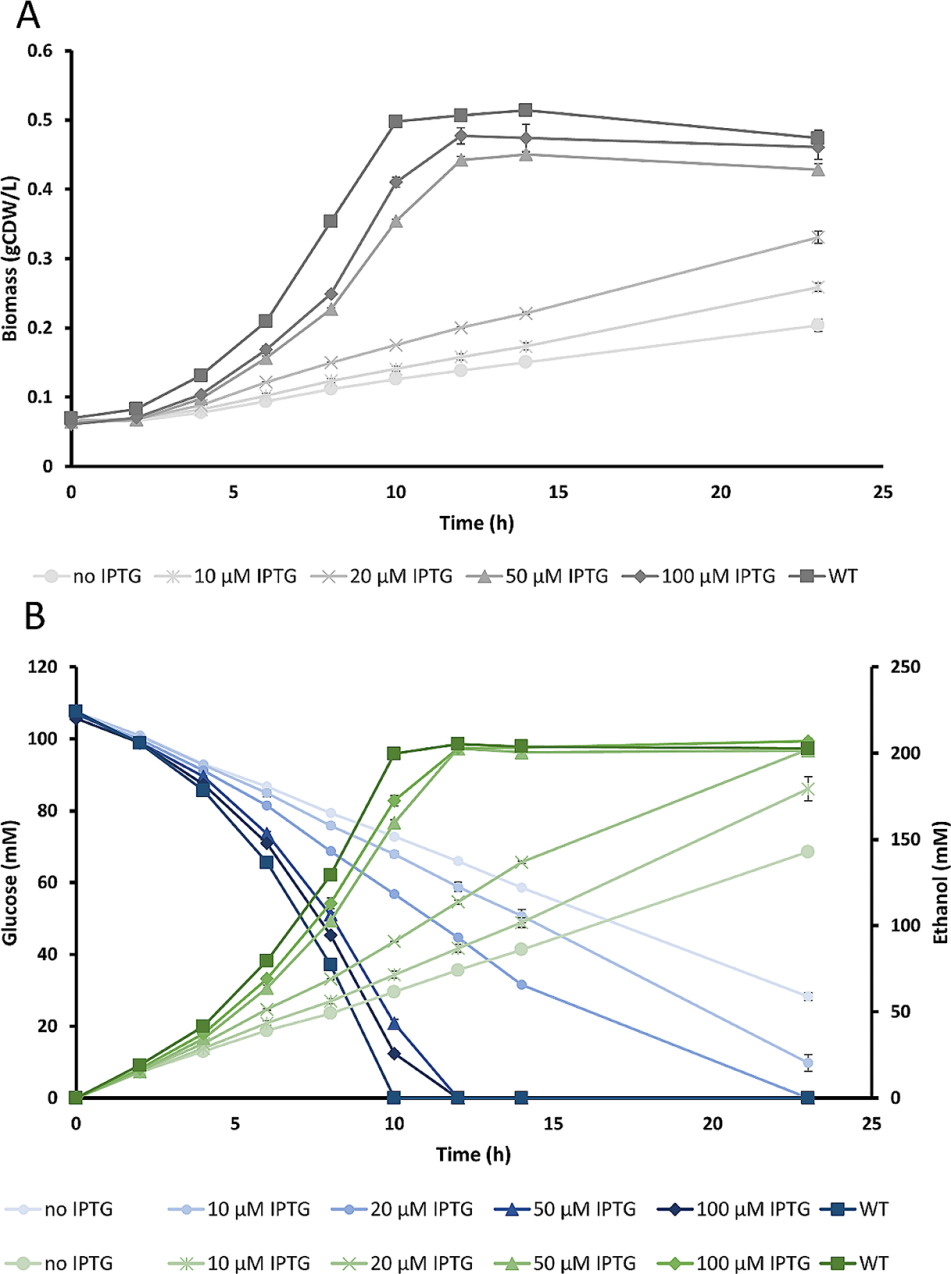
Growth of ZM4 and sGB027 in minimal medium (ZMM) with varying IPTG concentrations. **A**: biomass formation and **B**: time course of ethanol (green) and glucose concentrations (blue). Error bars represent the standard deviations of 3 biological replicates

To assess the growth behavior of strain sGB027 in dependence of the amount of added IPTG, we conducted anaerobic growth experiments in minimal medium with 2% glucose (111 mM) and varying IPTG concentrations (Fig. 2; Table 1). We observed that growth rate as well as the final biomass increased with increasing IPTG concentrations (Fig. 2). While growth was severely constrained when no inducer was added to the medium (Table 1), some ethanol was still produced and ethanol yields from glucose remained constant and similar to ZM4, most likely due to a certain leakiness of the P_T7A1_ promoter system, which was also observed by Liu et al. [15]. We analyzed *pdc* expression as well as PDC activity from these cultures after growing for 6–7 h. Both PDC activity as well as expression showed a gradual response to the amount of IPTG reflecting the observed growth rates. Without IPTG, PDC activity as well as expression were strongly reduced to about 1–7% of the ZM4 level (see Additional File, Fig. S1).

### Lactate production with sGB029

**Fig. 3:**
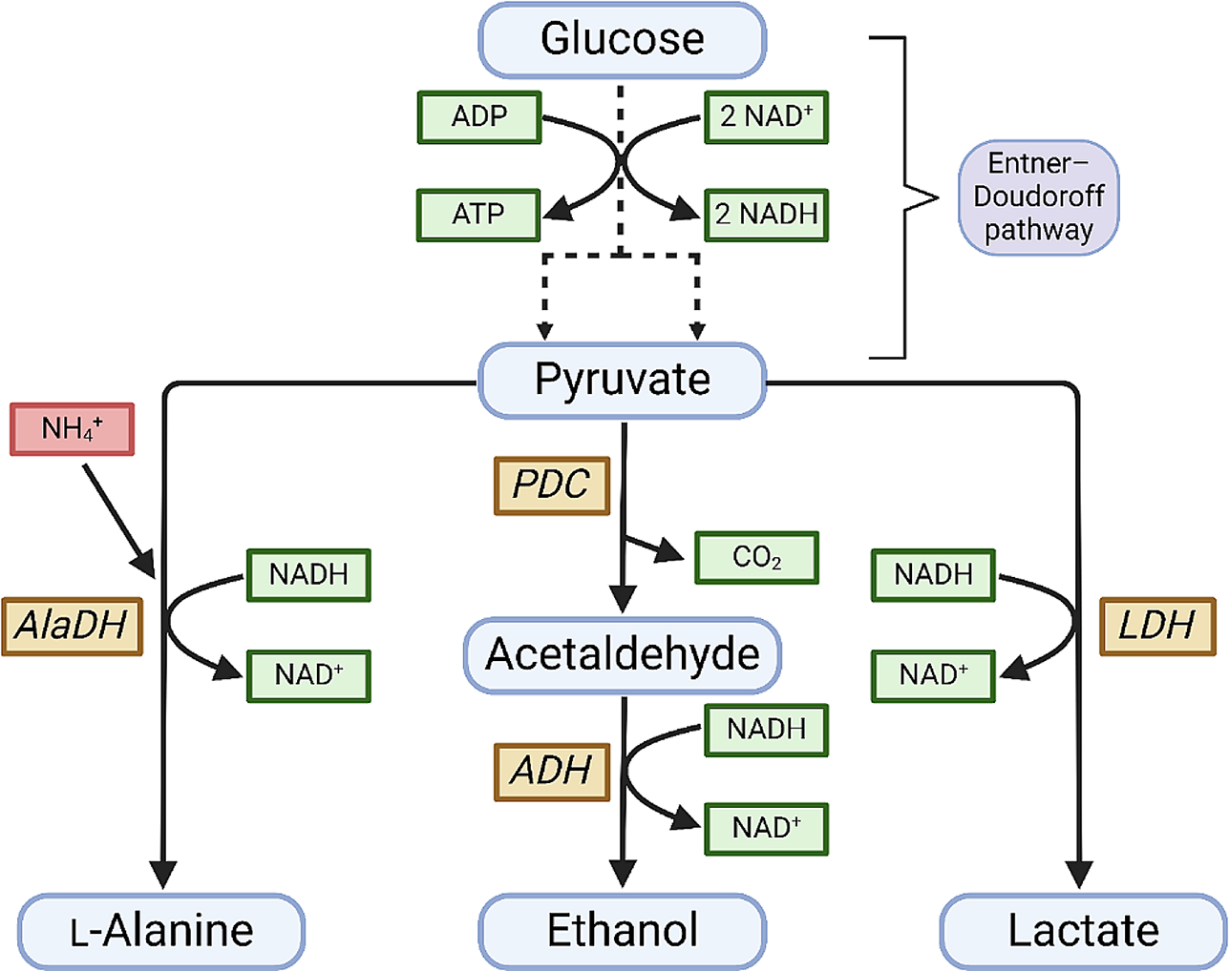
Metabolic pathways branching from pyruvate. PDC, pyruvate decarboxylase; LDH, lactate dehydrogenase; ADH, alcohol dehydrogenase; AlaDH, alanine dehydrogenase. Created with BioRender.com.

Growth deficiency of sGB027 at low IPTG concentrations is most likely a result of the reduced flux from pyruvate to ethanol due to limited PDC availability. As pyruvate production is not redox balanced, the glycolytic flux cannot be maintained due to missing alternatives to recycle NAD^+^ by oxidation of NADH. Therefore, we investigated if an alternative redox-balanced pathway from pyruvate could enhance cell growth. The formation of two molecules of lactate from glucose has the same neutral NADH balance as the formation of ethanol (Fig. 3) and lactate could thus replace ethanol as fermentation product. We therefore transformed sGB027 with plasmid pZP536 carrying the *ldhA* gene (lactate dehydrogenase) of *E. coli* under control of the constitutive strong promoter P_strong100k*_ [9] to create sGB029.

**Fig. 4:**
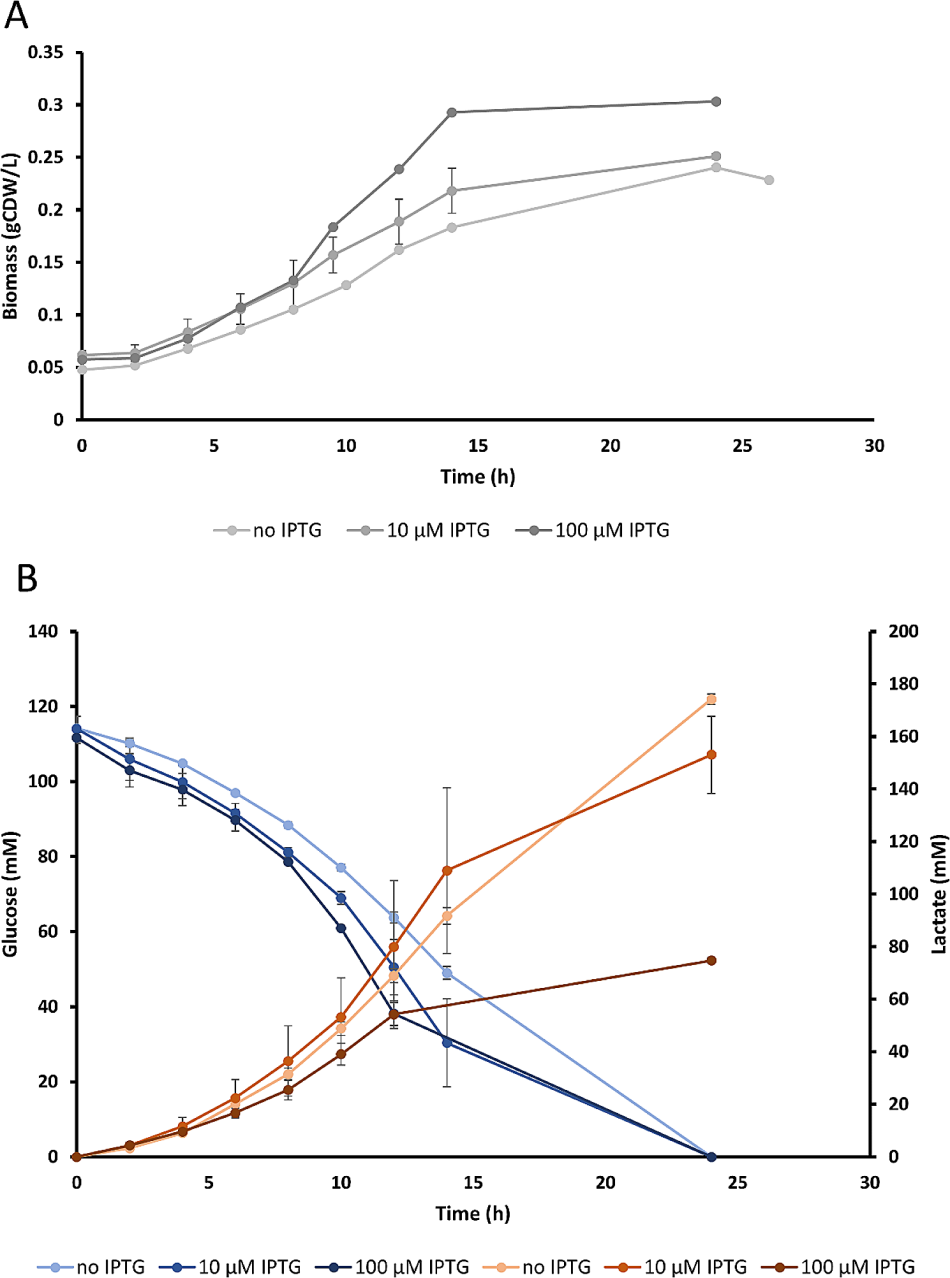
Growth of sGB029 in minimal medium (ZMM) with varying concentrations of IPTG. **A**: Biomass formation and **B**: glucose consumption (blue) and lactate production (red) of sGB029 in dependency of IPTG concentration. Error bars represent the standard deviations of 3 technical replicates (no IPTG and 100 µM IPTG) or 3 technical replicates of 2 independent experiments (10 µM IPTG), respectively

Under anaerobic conditions in chemically defined medium (ZMM), sGB029 exhibited a growth behavior similar to sGB027 when expressing *pdc* with increasing IPTG concentrations, however, at low IPTG concentrations growth of sGB029 was enhanced compared to sGB027, showing that expression of LDH and hence an alternative NAD^+^ providing pathway can partially restore growth (Figs. 2 and 4; Table 1). IPTG concentrations showed a positive correlation to the specific glucose uptake and specific ethanol production rates, while specific lactate production showed a negative correlation to the IPTG concentrations (Table 1). Lactate production in chemically defined medium with 2% glucose (111 mM) without IPTG in a fermenter system yielded 173.6 ± 2.2 mM lactate (78% of the theoretical maximum) with a remarkable specific productivity of 66.6 ± 0.6 mmol gCDW^− 1^ h^− 1^ while ethanol production was strongly reduced to 18% of the theoretical maximum (Table 1). Glucose was fully depleted and the glucose uptake rate of 47.36 ± 0.04 mmol gCDW^− 1^ h^− 1^ was still 89% of that of the wild-type strain (Table 1). To be able to compare our strain to the lactate producing strain of Liu et al. [15] (cultivated in rich medium with glucose (RMG)), we conducted a fermentation in complex medium (ZCM) reaching an even higher specific lactate productivity of 92.45 ± 4.75 mmol gCDW^− 1^ h^− 1^ and a higher yield of 81% of the theoretical maximum (Table 1; note that in this fermentation the glucose start concentration was only 100 mM compared to 111 mM in defined medium explaining the slightly lower lactate titer).

**Table 1:** Characteristics of strains sGB027 and sGB029. Growth rate, glucose uptake, and production of ethanol, lactate and alanine of strains WT (ZM4), sGB027 and sGB029. µ: growth rate; *r*: specific uptake/production rates; *Y*: yields; *P*: final product titers; n.d. not detected. Values and standard deviations are derived from either 3 biological replicates (WT and sGB027) or from 2 independent experiments each with 3 technical replicates.^1)^ conducted in undefined complex medium (ZCM). ^2)/3)^ conducted in ZMM with 10% glucose (^2)^ 0–23.5 h, ^3)^ 23.5–28.5 h), values and standard deviations are derived from 2 independent experiments. Yields and final product titers were determined after 28.5 h and hence integrate the results from both phases from 0 to 28.5 h

**Strain**	IPTG (µM)	µ (1/h)	*r* (mmol gCDW^− 1^ h^− 1^)			*Y* (mol/mol Glucose)		*P* (mM)	
			*r*_Glucose_	*r*_Ethanol_	*r*_Lactate_	*Y*_Ethanol_	*Y*_Lactate_	*P*_Ethanol_	*P*_Lactate_
WT	0	0.224 ± 0.003	53.40 ± 0.83	96.48 ± 1.22	n.d.	1.90 ± 0.03	n.d.	205.4 ± 1.8	n.d.
sGB027	0	0.054 ± 0.002	25.70 ± 0.61	45.14 ± 0.92	n.d.	1.81 ± 0.01	n.d.	142.9 ± 1.61	n.d.
	10	0.061 ± 0.001	26.51 ± 0.65	47.82 ± 1.56	n.d.	1.83 ± 0.03	n.d.	179.3 ± 6.9	n.d.
	20	0.075 ± 0.000	39.37 ± 0.27	69.86 ± 0.95	n.d.	1.89 ± 0.02	n.d.	202.4 ± 1.4	n.d.
	50	0.188 ± 0.000	49.73 ± 0.73	94.08 ± 1.39	n.d.	1.89 ± 0.02	n.d.	202.8 ± 2.17	n.d.
	100	0.192 ± 0.003	46.58 ± 1.00	88.53 ± 0.57	n.d.	1.91 ± 0.02	n.d.	202.8 ± 0,5	n.d.
sGB029	0	0.089 ± 0.002	47.36 ± 0.04	18.45 ± 0.40	66,67 ± 0.62	0.35 ± 0.01	1.53 ± 0.01	40.2 ± 0.8	173.6 ± 2.2
	10	0.103 ± 0.002	47.91 ± 4.47	26.85 ± 3.79	64,86 ± 9.66	0.54 ± 0.12	1.39 ± 0.03	61.0 ± 11.7	153.0 ± 14.7
	100	0.134 ± 0.000	47.08 ± 1.59	56.50 ± 8.07	32.27 ± 4.73	1.29 ± 0.03	0.67 ± 0.02	137.6 ± 7.8	74.7 ± 0.9
sGB029^1^	0	0.136 ± 0.005	53.43 ± 0.67	13.62 ± 0.79	92.45 ± 4.75	0.32 ± 0.02	1.62 ± 0.04	30.9 ± 1.9	161.7 ± 10.8
sGB029^2^	0	0.082 ± 0.000	65.33 ± 0.58	20.20 ± 1.23	93.45 ± 0.06	0.31 ± 0.02	1.43 ± 0.01	111.71 ± 4.88	511.65 ± 14.67
sGB029^3^	0	-	7.31 ± 0.46	2.31 ± 0.25	11.80 ± 0.76				

To test how higher concentrations of the substrate glucose affect lactate production, we performed additional bioreactor experiments in ZMM with 10% glucose. While the specific glucose uptake rate (65.3 ± 0.6 mmol gCDW^− 1^ h^− 1^) and lactate production rate (93.5 ± 0.1 mmol gCDW^− 1^ h^− 1^) within the first 23.5 h was significantly enhanced when compared to the cultivation with 2% glucose, a stronger reduction could be observed after 23.5 h for both, the glucose uptake rate (7.3 ± 0.5 mmol gCDW^− 1^ h^− 1^) and the lactate production rate (11.8 ± 0.8 mmol gCDW^− 1^ h^− 1^) (Table 2). The cells were still producing lactate after 23.5 h and reached a titer of 46 g/L (511 mM) after 28.5 h while 2% of the initial glucose were still in the medium. We hypothesize that the high concentration of lactate at the end of the fermentation might have inhibitory effects on growth and substrate uptake.

### Alanine production with sGB038

**Fig. 5:**
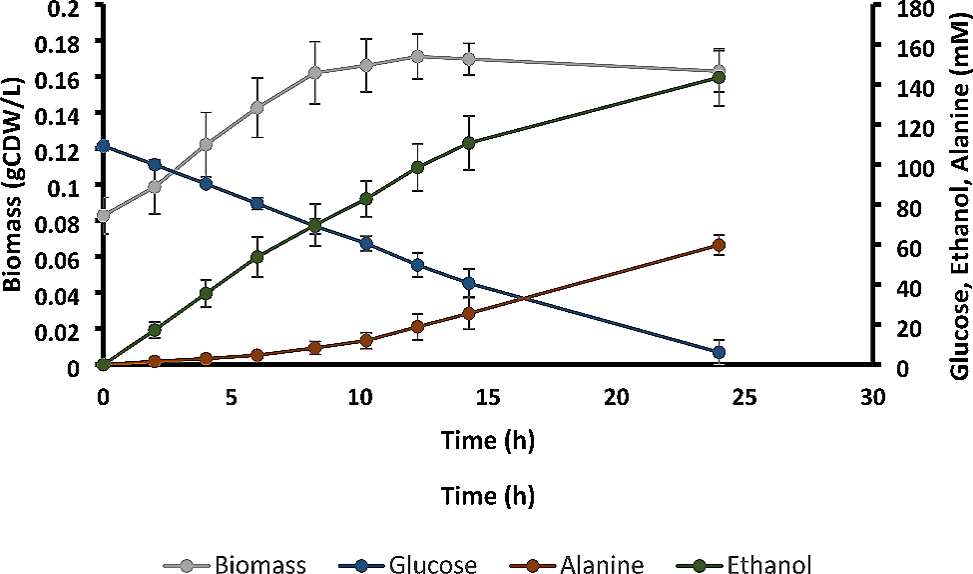
Fermentation of sGB038 in minimal medium (ZMM) with ammonium spikes. Biphasic growth behavior of Z. mobilis sGB038 (grey), glucose consumption (blue), ethanol (green) and alanine (red) production. Error bars represent the standard deviations of 2 representative independent fermentations with 3 technical replicates each

To assess whether strain sGB027 can also be used to produce other compounds derived from pyruvate, we aimed for production of L-alanine by a strategy similar to Uhlenbusch et al. [12] by expressing a codon-optimized alanine dehydrogenase (AlaDH) of *Geobacillus stearothermophilus* in sGB027, resulting in sGB038 (see Material and Methods). As for ethanol and lactate, the pathway from glucose to alanine is redox-balanced (Fig. 3), however, for the production of alanine from pyruvate, one molecule of ammonium needs to be provided for each molecule of alanine to be produced. Hence high amounts of ammonium have to be supplied. Therefore, we tested different ammonium sources for their utility for *Z. mobilis*. Di-ammonium phosphate, di-ammonium sulfate, and ammonium chloride led to a growth inhibition at concentrations of 50 mM, 50 mM, and 100 mM, respectively (Supplemental File 1, Fig S2). Therefore, we decided to apply a fermentation strategy with spiking ammonium chloride (to which *Z. mobilis* showed the highest tolerance) to alleviate harmful effects of ammonium on the cell growth of *Z. mobilis*. Using this setup in a cultivation without induction of *pdc*, led to a titer of 58.76 ± 4.27 mM alanine after 24 h of cultivation (Table 2; note that the total amount of ammonium provided would have allowed for a maximum production titer of alanine of 102.5 mM). Notably, we observed a biphasic growth and production behavior for sGB038 (Fig. 5). In the first phase, which lasted about 8–10 h after inoculation, depending on initial biomass, we observed a linear growth behavior and a relatively low specific alanine productivity (9.8 ± 2.4 mmol gCDW^− 1^ h^− 1^) coupled to a corresponding higher specific productivity of ethanol (70.3 ± 5.4 mmol gCDW^− 1^ h^− 1^). In the second phase, growth stopped but alanine productivity was enhanced (18.8 ± 3.0 mmol gCDW^− 1^ h^− 1^) while ethanol production was significantly reduced (32.5 ± 6.9 mmol gCDW^− 1^ h^− 1^). We hypothesized that the observed biphasic behavior of sGB038 is caused by residual PDC present in the cells transferred from the seed culture where *pdc* expression was induced with 50 µM IPTG. We therefore measured the PDC activity at different time points. At the start of the experiment, we detected a PDC activity of 2.7 pmol µg_Protein_^−1^ sec^− 1^ and the activity decreased to 2.1 and 0.8 pmol µg_Protein_^−1^ sec^− 1^ after 4 and 11 h, respectively (Additional File 1, Fig. S1l. Apparently, the strongly reduced PDC activity after 11 h resulted in growth arrest and a doubled alanine production rate (Table 2).

**Table 2:** Growth rate, glucose uptake, ethanol and alanine production of strain *sGB038* (IPTG was not added in this experiment). ${\mu }_{lin}$: linear growth rate; *r*: specific uptake/production rates; *Y*: final yields; *P*: final product titers. The first phase was production phase with linear growth and the second phase production without growth (see Fig. 5). Data are derived from 3 technical replicates of 3 independent experiments

Phase	${\mu }_{lin}$ (gCDW/h)	*r* (mmol gCDW^− 1^ h^− 1^)			*Y* (mol/mol Glucose)		*P* (mM)	
		*r*_Glucose_	*r*_Ethanol_	*r*_Alanine_	*Y*_Ethanol_	*Y*_Alanine_	*P*_Ethanol_	*P*_Alanine_
1	0.074 ± 0.008	40.53 ± 3.21	70.30 ± 5.40	9.83 ± 2.40				
2	0	27.19 ± 1.89	32.53 ± 6.91	18.75 ± 3.00				
total					1.28 ± 0.16	0.63 ± 0.09	122.3 ± 32.2	58.8 ± 4.3

## Discussion

Even though there have been many attempts to delete *pdc* in the WT *Z. mobilis* background, none has been successful so far. The essentiality of the PDC for a functioning metabolism paired with the polyploidy exhibited by *Z. mobilis* [30–32] render a complete removal of *pdc* hitherto impossible. The only successful complete removal was reported in a patent [18]. Here, after introduction of the heterologous pathway for 2,3-butanediol production, an attempt to knockout *pdc* resulted in a strain with both the modified as well as the engineered allele. After several culture transfers a clean knockout with high product yields could be obtained. However, to our knowledge, this approach has not been reported so far in peer review publications and it does not easily allow a switch to another product pathway as is possible with our platform strain sGB027. A knockdown or partial knockout however already shows beneficial effects on the yields of products other than ethanol [8, 14–17]. The most promising approach was presented by Liu et al. [15] who expressed *pdc* of *Z. mobilis* from a plasmid and subsequently deleted the original *pdc* locus (ZMO1360) from the chromosome. Low level expression of the controlled *pdc* copy paired with high level expression of genes for alternative product pathways, resulted in strongly increased yields of heterologous products like 2,3-BDO, isobutanol and lactate [15]. We followed a similar but different approach by directly replacing the native *pdc* promoter (317 bp) in the chromosome with an IPTG-controlled promoter. This avoids possible issues related to plasmid stability and makes addition of antibiotics unnecessary. Furthermore, plasmid-based expression of genes is difficult to fine-tune and could vary with growth-phase and environmental conditions. In addition, the plasmid-based expression of *pdc* restricts the expression of heterologous genes, as compatible plasmids have to be found that stably coexist in *Z. mobilis*. Also, two or even three plasmid strategies would demand to find two or three selection markers that can be combined without side effects on cell growth or selection strength. Until recently, compatible replication systems for *Z. mobilis* were also not widely available [33]. Our strain sGB027 is based on chromosomal manipulation that promises to be more stable and simplifies the introduction of additional genes using expression plasmids, creating a platform-strain that can easily be further modified by addition of different heterologous genes. sGB027 showed a strong growth impairment when no inducer was added (Fig. 2). To rescue the growth phenotype, which is most likely due to a redox imbalance caused by a depleted NAD^+^ pool, and to couple growth to lactate production we introduced the lactate dehydrogenase gene *ldhA* of *E. coli* on an expression plasmid under the control of the strong constitutive promoter P_strong100k*_ [9] resulting in strain sGB029. We chose lactate because it is already widely used in the chemical and food industry as well as in the cosmetic and the pharmaceutical industry and is currently mainly produced by microbial processes [34, 35].

To the best of our knowledge, our new strain sGB029 was able to achieve the highest specific lactate productivity (66.7 ± 0.6 mmol gCDW^− 1^ h^− 1^ in defined minimal medium, 92.5 ± 4.8 mmol gCDW^− 1^ h^− 1^ in complex medium with 2% glucose, and even 93.45 ± 0.1 mmol gCDW^− 1^ h^− 1^ in defined minimal medium with 10% glucose) and also outperforms other *Z. mobilis* strains [14, 15] (specific productivities estimated from experimental data in these references). Moreover, our strain performed also better in terms of yields in both minimal (1.53 mol/mol glucose; Table 1) and undefined complex medium (1.62 mol/mol glucose) compared to the strain of Liu et al. (about 1.40 mol/mol glucose) cultivated in rich medium. Generally, the use of complex additives such as yeast extract come with significantly increased costs that would be prohibitive for industrial production of high-volume and low-cost chemicals [36]. Ideally, low-cost media would be chosen for industrial production, e.g. based on residual streams such as lignocellulose hydrolysates or molasses. In our future work, we will investigate whether our strain achieves in suitable low-cost media a similarly good performance as observed in both complex and chemically defined medium.

Hu et al. [14] followed a similar genetic strategy as in our approach and integrated *pdc* expressed from an inducible promoter into an alternative chromosomal locus. They tested both, integration of a heterologous lactate dehydrogenase into the chromosome as well as expression from a plasmid. However, they were only able to achieve lactate yields of 0.98 mol/mol glucose even in undefined complex media compared to the 1.62 mol/mol glucose in our approach.

As another showcase of the applicability of sGB027 as a platform strain, we established L-alanine production by expressing the alanine dehydrogenase of *G. stearothermophilus* in our *pdc* mutant sGB027. We were able to produce 58.8 ± 4.3 mM alanine from 94.0 ± 14.5 mM of glucose. Even without further optimization, our strain is already able to achieve a similar specific productivity as the *E. coli* strain XZ132, which is used for industrial production of alanine (1.7 ± 0.3 g g^− 1^ h^− 1^ for our strain vs. 1.98 g g^− 1^ h^− 1^ for the *E. coli* strain) [37, 38]. While the yield of our strain (0.63 mol/mol glucose) is below the high alanine yield of the *E. coli* producer strain (1.90 mol/mol glucose), we believe that optimization of alanine production can lead to yields which are comparable to the lactate yields of sGB029.The biphasic behavior of sGB038 regarding alanine production might be caused by residual PDC still being active in the cells in the first 8–10 h, resulting in growth and a relatively high ethanol production. Since this behavior was not observed in the lactate producing strain, it could also be caused by growth inhibition caused either by alanine itself or by the addition of ammonium chloride. The growth behavior could also be linked to a low activity of AlaDH, that might be caused by a suboptimal pH value within the cell, since the pH optimum of the *G. stearothermophilus* AlaDH for the reductive amination of pyruvate is at 9.5 [39], while the intracellular pH of anaerobically growing *Z. mobilis* cells is at around 5.5 [40]. Another factor limiting alanine production might be its low excretion rate observed in *Z. mobilis* [12]. The biphasic behavior could be exploited for an intentionally designed two-stage process, where the cells can grow to sufficiently high biomass concentrations in the first stage followed by an alanine production phase where *pdc* activity is strongly reduced. A similar approach could be used for the lactate producer strain. To make such two-stage processes more cost-efficient, it would then be necessary to replace the IPTG-based system with other suitable promoters, e.g. temperature-dependent promoters or AHL-mediated quorum-sensing circuits [41, 42]. We see sGB027 as a platform strain that will need further specific optimizations for industrial applications, for example, the extension of its substrate spectrum or an increased tolerance towards inhibitory substances that might be present in the medium of choice. Although we did not observe mutants of our strain that escape the IPTG control of *pdc*, for some processes it might be necessary to increase its stability e.g. by additional copies of *lacI* or by applying a similar control to the alcohol dehydrogenases.

## Conclusion

In this study we presented a new platform strain of *Z. mobilis* in which the native promoter of *pdc* was replaced with an IPTG-inducible promoter, allowing for a controllable expression of *pdc*. Based on this strain we built two other strains dedicated to lactate and alanine production, respectively. In particular, the lactate producer strain synthesized lactate with unprecedented specific productivity and with high yield. These results demonstrate the potential of sGB027 as a platform strain for various applications and the suitability of our approach for controlled redirection of carbon flux around the pyruvate node in *Z. mobilis*.

### Electronic supplementary material

Below is the link to the electronic supplementary material.

Additional file 1: Plasmids, strains and primer used in this study. Annotated gene bank files of all related DNA sequences as in an online repository (https://doi.org/10.17617/3.UM0Q7A).

## Data Availability

Data is provided within the manuscript or supplementary information files.

## Abbreviations

PDC: Pyruvate decarboxylase
GRAS: Generally regarded as safe
ZMM: Zymomonas minimal medium
ZCM: Zymomonas complex medium
*ldhA*: lactate dehydrogenase of *Escherichia coli*
*AlaDH*: Alanine dehydrogenase of *Geobacillus stearothermophilus*
