# Supplementary material for: A new Zymomonas mobilis platform strain for the efficient production of chemicals
Source: Microb Cell Fact. 2024 May 22;23:143. doi: 10.1186/s12934-024-02419-9 (PMC11110354; doi:10.1186/s12934-024-02419-9)
Supplement: Supplementary file 1 — Additional file 1: Plasmids, strains and primer used in this study. Annotated gene bank files of all related DNA sequences as in an online repository (https://doi.org/10.17617/3.UM0Q7A). [file 12934_2024_2419_MOESM1_ESM.docx]

**Additional File**

**A new *Zymomonas mobilis* platform strain for the efficient production of chemicals**

Jonas Frohwitter^1,a^, Gerrich Behrendt^1,a^, Steffen Klamt^1^, Katja Bettenbrock^1, *^

^1)^ Analysis and Redesign of Biological Networks, Max Planck Institute for Dynamics of Complex Technical Systems, Sandtorstr. 1, 39106, Magdeburg, Germany

^a)^ These authors contributed equally.

^*^Corresponding author: bettenbrock@mpi-magdeburg.mpg.de

**Table S1:** Plasmids used in this study.

| **Plasmid** | **Description** | **Assembly** |
| --- | --- | --- |
| pZP014 | lvl1-pos2-accceptor | [1] |
| pZP016 | lvl1-pos4-accceptor | [1] |
| pZP017 | lvl1-pos5-accceptor | [1] |
| pZP027 | lvl1-pos1-dummy | [1] |
| pZP038 | lvl2-end-linker-5 | [1] |
| pZP131 | lvl1-pos3rev-SpecR_cassette | [1] |
| pZP137 | lvl2-pos1-acceptor-suicide | [1] |
| pZP159 | rbs-10k | [1] |
| pZP286 | lacI-PlacT7A1-in-001 | [1] |
| pZP289 | terminator-of-soxR-in-004 | [1] |
| pZP436 | Pstrong100k*-in-001 | [1] |
| pZP536 | lvl1-pos4-Pstrong100k*-ldhA-TrrnB1 | [1] |
| pZP941 | lvl1-pos4-lacI-PlacT7A1-for-GE | Pr 890 + Pr 891 (Templ. pZP286 , Atemp. 60 °C, 1700 bp) BsaI Cut-Ligation into pZP016 |
| pZP948 | lvl1-pos2-usHA-pdc-promoter-switch | Pr 900 + Pr 901 (Templ. gDNA ZM4 , Atemp. 60 °C, 650 bp) BsaI Cut-Ligation into pZP014 |
| pZP949 | lvl1-pos5-dsHA-pdc-promoter-switch | Pr 902 + Pr 903 (Templ. gDNA ZM4 , AT 60 °C, 100 bp),  Pri 904 + Pr 905 (Templ. gDNA ZM4 , AT 60 °C, 700 bp) BsaI Cut-Ligation into pZP017 |
| pZP950 | lvl2-pos1-GE-pdc-promoter-switch-SpecR-lacI-PlacT7A1-suicide | pZP137 + pZP027 + pZP948 + pZP131 + pZP941 + pZP949 + pZP038 BbsI Cut-Ligation |
| pZP1254 | alaD-Geobacillus_stearothermophilus-pos003v2 | Synthesized by TWIST Biosciences, codon optimisation performed based on KAZUSA |
| pZP1257 | lvl1-pos4-Pstrong100k*-alaD-G_stearothermophilus-TsoxR | pZP016 + pZP436 + pZP159 + pZP1254 + pZP289 BsaI Cut-Ligation |

Pr: Primer, [1] Behrendt et al., 2022 ACS Synthetic Biology 11(11), 3855-3864 DOI: 10.1021/acssynbio.2c00428

**Tab. S2:** Strains used in this study.

| **Strain** | **Base** | **Genotype** | **Plasmid /Enzyme expressed** |
| --- | --- | --- | --- |
| ZM4 |  |  |  |
| sGB027 | ZM4 wt | ZM4 ΔPpdc :: SpecR-lacI-PlacT7A1 | pZP950 (suicide) |
| sGB029 | sGB027 | ZM4 ΔPpdc :: SpecR-lacI-PlacT7A1/ pZP536 | pZP536 / LdhA |
| sGB038 | sGB027 | ZM4 ΔPpdc :: SpecR-lacI-PlacT7A1 /pZP1257 | pZP1257 / AlaDH |

**Tab. S:3** Primer used in this study.

| **Primer** | **Sequence (5´...3´)** |
| --- | --- |
| Primer 890 | ggaGGTCTCaAGTGggtctgcgttgtcg |
| Primer 891 | ggaGGTCTCaGGTTaattgttatccgctcacaattgaatctaagtatca |
| Primer 900 | ggaGGTCTCaAGTGCACAATAATGGCAAAGCTGCTGACA |
| Primer 901 | ggaGGTCTCaGGTTGCCTGTTTTAAAATTTTTCCGGCTGTT |
| Primer 902 | ggaGGTCTCaAGTGGGGTCATCCTGATTCAGACATAGTG |
| Primer 903 | ggaGGTCTCaGGCCAATCTGGACAAGCCGCTCCG |
| Primer 904 | ggaGGTCTCaGGCCTCAAGCATCACTTCGCAGTCGC |
| Primer 905 | ggaGGTCTCaGGTTTGGGAAGAAGCTTTTTGCAGC |
|  |  |
| gap-for-real | ACCCCGGATGTCAGCCTTGTTG |
| gap-rev-real | GGAGCTGTGCGGATCGGAGTAGAA |
| zwf-for-real | AAGTTGCCGGTTACATTGACGA |
| zwf-rev-real | TGCGGATATAGAACGGAACACC |
| pdc-for-real | AGCTTCTTCCGGGGTGTAAATC |
| pdc-rev-real | GTGGCGCCTATGCAGAAAACCT |

**
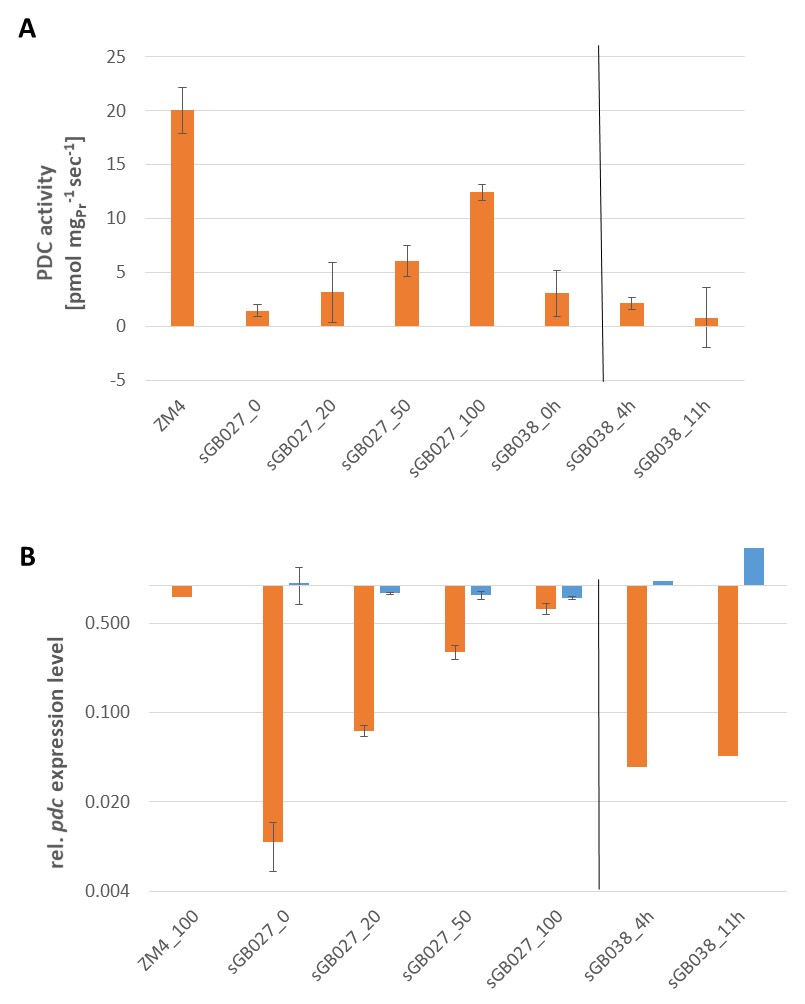
**

**Fig. S1:** **PDC activity and *pdc* expression levels in sGB027 in dependence of IPTG and in sGB038 fermentations at two different time points**. Data for sGB027 were derived from two independent growth experiments after approximately 6-7 hours of growth in ZMM when cultures showed a constant exponential growth rate. Numbers after the strain name sGB027 indicate the amount of IPTG added in µM. PDC activity for sGB038 were derived from two independent growth experiments after 4h and 8h, respectively, with no IPTG added. Data for *pdc* expression from sGB038 are determined from one experiment only. **A)** PDC activity in pmol µg_Protein_^-1^ sec^-1^. **B)** Gene expression analysis by Real Time RT-PCR evaluated by the ΔΔCT method using the genes *gap* and *zwf* as housekeeping genes and ZM4_0 as reference sample. Data are plotted on a logarithmic scale to better visualize the changes. ZM4_100 is included to show that IPTG has no effect in *pdc* expression in the WT (data from one run only). We also show data for *glk* (in blue) to demonstrate the variation of a gene that is not controlled by IPTG.

**Fig. S2:** **Growth of ZM4 WT in ZMM (2% glucose) with different nitrogen sources.** Data were derived from two biological replicates. Control: ZMM with 1g/L (7.6 mM) Ammonium sulfate. Nitrogen sources were added at the start of the cultivation.
